# Re-emerging outbreaks of chikungunya virus infections of increased severity: A single-center, retrospective analysis of atypical manifestations in hospitalized children during the 2019 outbreak in Bangkok, Thailand

**DOI:** 10.1371/journal.pone.0330527

**Published:** 2025-09-25

**Authors:** Artchavit Boonanek, Kulkanya Chokephaibulkit, Wanatpreeya Phongsamart, Keswadee Lapphra, Supattra Rungmaitree, Navin Horthongkham, Orasri Wittawatmongkol

**Affiliations:** 1 Division of Infectious Diseases, Department of Pediatrics, Faculty of Medicine Siriraj Hospital, Mahidol University, Bangkok, Thailand; 2 Siriraj Institute of Clinical Research (SICRES), Faculty of Medicine Siriraj Hospital, Mahidol University, Bangkok, Thailand; 3 Department of Microbiology, Faculty of Medicine Siriraj Hospital, Mahidol University, Bangkok, Thailand; Universidad Cooperativa de Colombia, COLOMBIA

## Abstract

This retrospective observational study assesses the clinical characteristics, atypical manifestations, and treatment outcomes in pediatric patients hospitalized with chikungunya virus (CHIKV) infection during the Bangkok outbreak in 2019. Children <18 years old hospitalized from January 1 to December 31, 2019, and confirmed positive for CHIKV infection by RT-PCR or IgM antibodies were included in this study at a tertiary care center in Bangkok, Thailand. Patient demographics, clinical manifestations, and laboratory findings at the time of hospitalization were collected from de-identified medical records. Of 31 included children, seven (22.6%) were <1 year old, 22 (71.0%) were male, nine (29.0%) had underlying medical conditions, four (17.4%) tested positive for dengue coinfection, and four (12.9%) had multi-organ involvement. The median age was 9.5 (IQR 6.9–12.5) years. Most (90.3%) had atypical clinical manifestations, four (12.9%) had life-threatening manifestations. Two (6.5%) neonates had congenital CHIKV. The most common manifestations included fever (100.0%), rashes (77.4%), myalgia (41.9%), and arthralgia (35.5%). The three most involved organ systems presenting atypical manifestations included gastrointestinal (32.3%), dermatologic (32.3%), and neurological (22.6%) systems. Of those with dermatologic involvement, 67.7% had maculopapular rashes, 19.4% bullous skin lesions, and 6.5% generalized erythroderma. At the time of presentation, 25 (80.6%) children had lymphopenia, five (16.1%) had anemia, and none had thrombocytopenia. Five (16.1%) children required intensive care and four (12.9%) developed shock. Thirteen (41.9%) children, five with neurological involvement, fully recovered at discharge. Among the remaining children, five (16.1%) still had musculoskeletal conditions, 11 (35.5%) had skin lesions, and two (6.5%) with congenital CHIKV had skin lesions and neurological sequelae. Despite the small cohort, the observed frequency of neurological complications attributed to CHIKV infection justifies long-term follow-up in children with neurological manifestations and complications. CHIKV should be suspected in endemic countries and tested for in febrile children, particularly those with rash and neurological involvement.

## Introduction

Chikungunya virus (CHIKV) is a mosquito-borne *Alphavirus* (family: Togaviridae) first identified in Tanzania, 1952. It subsequently caused outbreaks across Africa, Europe, the Americas, South and Southeast Asia, as well as Oceania [1]. CHIKV infections typically have self-limited presentations characterized by viral exanthematous fever (i.e., fever, headache, fatigue, and/or a maculopapular rash), polyarthralgia, and other systemic symptoms (i.e., myalgia, nausea, and/or vomiting) [2–5]. Atypical and severe, even fatal, manifestations of CHIKV as well as evidence of vertical transmission were first described in 2005–2006 during an outbreak on Réunion Island [6,7]. Recent studies revealed that younger children often presented with undifferentiated fever [8], were less likely to exhibit the typical CHIKV manifestations observed in older children, and instead presented with a bullous rash or neurological manifestations [9–11]. While the clinical manifestations of dengue virus, zika virus, and CHIKV infection are similar, the prevalence of these manifestations differs substantially overall, by age, and by day of illness [12,13].

The first documented CHIKV outbreak in Asia occurred in 1958 Bangkok, Thailand. Another large outbreak took place between 2008–2009 in Southern Thailand, during which a few atypical adult cases presenting with neurological manifestations were reported [14–16]. During an outbreak in Thailand in 2019, more than 13,000 cases of CHIKV were reported in Thailand, with a morbidity rate of 47.27 per 100,000 population, and more than 2,700 cases reported in Bangkok alone since the last outbreak more than 60 years ago.

Atypical manifestations and severe presentations from CHIKV have been reported in both children and adults in recent outbreaks [17]. Few studies have explored the clinical features of CHIKV infections in children, which may differ from adults. This single-center study assesses the clinical characteristics, atypical manifestations, and treatment outcomes in pediatric patients hospitalized with CHIKV infection during the Bangkok outbreak in 2019.

## Materials and methods

### Study design and population

This retrospective observational study described the clinical characteristics of atypical manifestations and treatment outcomes in pediatric patients hospitalized with CHIKV infection at Siriraj Hospital, the largest tertiary and quaternary care hospital during the Bangkok outbreak in 2019, Thailand. Inclusion criteria included being under 18 years old, hospitalized due to indications of CHIKV infection from January 1 to December 31, 2019, and confirmed positive for CHIKV infection by the detection of reverse transcription–polymerase chain reaction (RT-PCR) or IgM antibodies. This study was performed per the Declaration of Helsinki (revised in 2013) and approved by the Human Research Protection Unit, Faculty of Medicine Siriraj Hospital, Mahidol University, Thailand (COA No. 678/2020). The need for assent or consent of eligible patients and their legal guardians was waived by the Siriraj Ethics Committee due to all patient information being deidentified.

### Data collection

Patient records identified using ICD-10-CM code A92.0 were retrieved for analysis. All hospitalized patients had a confirmed diagnosis of CHIKV infection; none were excluded. Patient demographics (age, biological sex, underlying medical conditions) clinical manifestations, and laboratory findings at the time of presentation/hospitalization were collected from deidentified medical records from August 17, 2020 to August 31, 2022.

### Laboratory methods

CHIKV RNA was extracted from 200 µL of blood plasma using the magLEAD 12gC automated extraction platform (System Science Co., Ltd., USA; product code: I7912) and eluted in 100 µL aliquots. Genesig standard kits for CHIKV (Primerdesign Ltd., UK; catalogue no.: Path-CHIKV-standard) were used per the manufacturer’s protocol for *in-vitro* quantification of CHIKV RNA. CHIKV-specific IgM antibodies were detected for one specimen using an in-house enzyme-linked immunosorbent assay (ELISA) [18] at the National Institute of Health, Ministry of Public Health, Thailand.

### Viral genotyping

Nearly complete CHIKV genomes were obtained through next-generation sequencing; outputs were provided as FASTQ files. Reads were assembled using CLC Genomics Workbench (v22.0, QIAGEN); consensus sequences were exported as FASTA files. For phylogenetic analysis, sequences were aligned with global CHIKV strains and analyzed using Molecular Evolutionary Genetic Analysis (MEGA, v11) software using maximum-likelihood and neighbor-joining methods, supported by 1,000 bootstrap replicates. Branch lengths reflected nucleotide substitutions per site.

### Definitions

Vertical transmission was defined by CHIKV infection in a neonate within the first seven days of life (DOL) after the birth mother contracted the virus during the perinatal period. Typical CHIKV manifestations were defined by mild-to-moderate viral exanthems with fever, maculopapular rash, and arthralgia/arthritis. Atypical manifestations were defined by presentations that involved other organ systems (i.e., neurological, cardiovascular, ophthalmological, hepatic, renal, and/or respiratory) or skin lesions other than maculopapular rashes. Severe CHIKV presentations were defined as life-threatening dysfunction of at least one organ or organ system [19]. Meningoencephalitis was defined as inflammation of the brain and its meninges, and encephalopathy was defined as a clinical state of altered mental status with or without inflammation of brain parenchyma; both require the presence of altered consciousness lasting >24 hours [20].

### Data analysis

Analyses were performed using Microsoft Excel (Ver 16.29). Continuous data were reported as median and interquartile range (IQR) and categorical data were reported as numbers and percentages.

**Fig 1:**
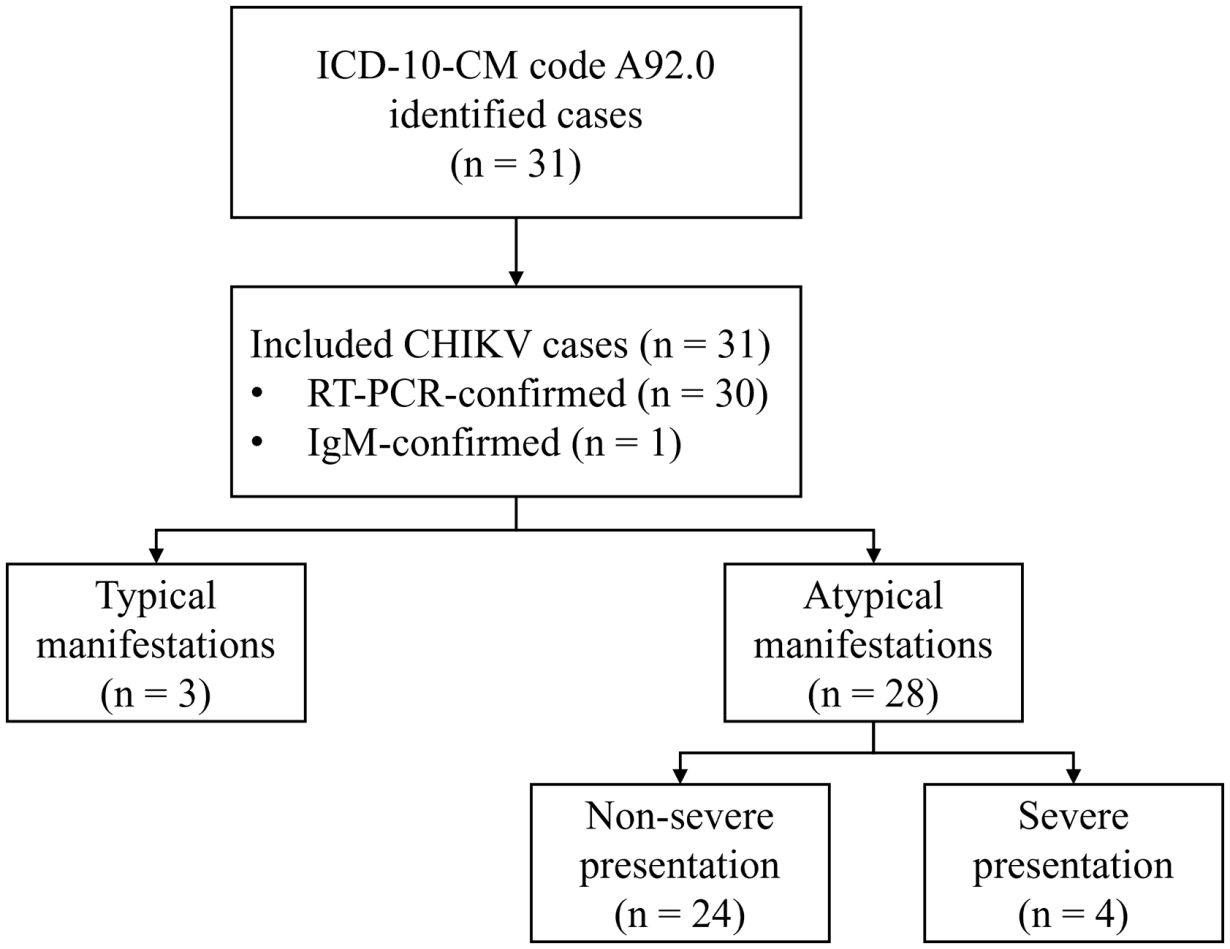
Flow diagram. Thirty-one children were included. Three children had typical manifestations, and 28 (90.3%) had atypical manifestations. Twenty-four children had non-severe presentations, and four had severe life-threatening presentations. Abbreviations: CHIKV, Chikungunya Virus.

## Results

### Patients

For the 31 included children, 30 cases were confirmed by serum/plasma CHIKV RT-PCR and one by positive IgM antibodies (Fig 1). Seven (22.6%) children were less than one year old (Table 1). Nine (29.0%) children had underlying medical conditions. Twenty-eight (90.3%) children had atypical clinical manifestations, and four (12.9%) had severe, life-threatening presentations. Two neonates had congenital CHIKV (6.5%). In the children tested for dengue coinfection (n = 23/31), 17.4% tested positive (n = 4/23), and none developed severe presentations or shock.

**Table 1:** Demographics, clinical manifestations, and laboratory findings of hospitalized children with Chikungunya Virus.

Characteristic	N = 31	
Age (y); median (IQR)	9.5 (6.9-12.5)	
Biological sex; n (%)		
Male	22 (71.0)	
Underlying medical conditions^a^; n (%)		
ASD	1 (3.2)	
Global developmental delay	1 (3.2)	
Renal disease(s)	2 (6.5)	
DM (Type I)	1 (3.2)	
MPHD	1 (3.2)	
Asthma	2 (6.5)	
OSA	1 (3.2)	
SLE	2 (6.5)	
Thalassemia	1 (3.2)	
DMD	1 (3.2)	
Neuropsychiatric disorders	2 (6.5)	
Atopic dermatitis	1 (3.2)	
**Clinical manifestations**	**N = 31**	
Fever; n (%)	31 (100.0)	
Duration (d); median (IQR)	3 (3-8)	
Biphasic fever; n (%)	5 (16.1)	
Musculoskeletal; n (%)	18 (58.1)	
Myalgia	13 (41.9)	
Arthralgia	11 (35.5)	
Arthritis	2 (6.5)	
Neurological; n (%)	7 (22.6)	
Headache	1 (3.5)	
Meningoencephalitis	5 (16.1)	
Encephalopathy	2 (6.9)	
Fever-associated seizure	3 (9.7)	
Hypotonia	1 (3.5)	
Dermatologic; n (%)	24 (77.4)	
Maculopapular	21 (67.7)	
Bullous	6 (19.4)	
Generalized erythroderma	2 (6.5)	
Cardiovascular; n (%)	4 (12.9)	
Septic shock	4 (12.9)	
GI; n (%)	10 (32.3)	
Nausea/vomiting	7 (22.6)	
Bleeding	2 (6.5)	
Respiratory; n (%)	3 (9.7)	
URT (rhinorrhea and cough)	2 (6.5)	
Pneumonia	1 (3.2)	
Hematologic; n (%)	2 (6.5)	
Petechiae	2 (6.5)	
Renal; n (%)	7 (22.6)	
AKI	7 (22.6)	
Rhabdomyolysis	1 (3.2)	
Ocular; n (%)	2 (6.5)	
Conjunctivitis	2 (6.5)	
Blepharitis	1 (3.2)	
**Laboratory findings at presentation; median (IQR)**	**Patient values** **(N = 31)**	**Reference** **Values** ^**b**^
Hb (g/dL)	12.7 (11.5-13.1)	11.5-15.5
WBC (cells/uL)	7,930 (5,105−9,785)	4,500−13,500
Absolute lymphocyte count (cells/uL)	976 (663−1,418)	1,500−7,000
Platelet count (cells/uL)	259,000 (191,500−288,500)	150,000-350,000
	n = 28	
GFR (mL/min)	107.0 (84.9-119.0)	>90
	n = 21	
AST (U/L)	46 (31-88)	18-36
ALT (U/L)	22 (16-41)	9-25

Abbreviations: GFR, Glomerular Flow Rate; DMD, Duchenne Muscular Dystrophy; OSA, Obstructive Sleep Apnea; ASD, Atrial Septal Defect; MPHD, Multiple Pituitary Hormone Deficiency; DM, Diabetes Mellitus; SLE, Systemic Lupus Erythematous; AKI, Acute Kidney Injury; URT, Upper Respiratory Tract; AST, Aspartate Aminotransferase; ALT, Alanine Aminotransferase; GI, Gastrointestinal.

^a^The remaining children had no underlying medical conditions.

^b^Reference values correspond to the age-specific IQR of children in this study per the Harriet Lane Handbook [21].

### Clinical manifestations

**Fig 2:**
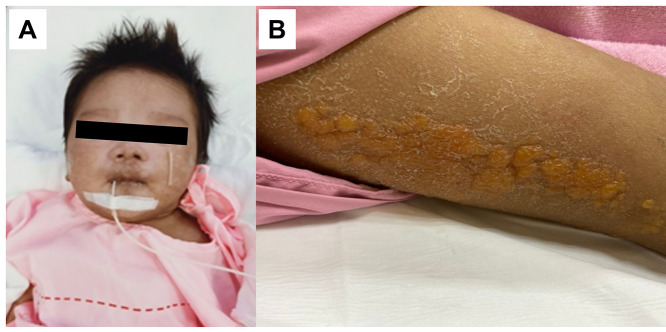
Skin manifestations of CHIKV infection in children. Hyperpigmentation in the centrofacial area of a neonate with congenital CHIKV (A) and bullous lesions along the right thigh of a 12-year-old girl (B).

Five children had biphasic fevers (Table 1). The most common manifestations included fever, rashes, myalgia, and arthralgia. Four (12.9%) children had multi-organ involvement. Of those with dermatologic involvement, 67.7% had maculopapular rashes, 19.4% bullous skin lesions (Fig 2), and 6.5% generalized erythroderma. Hyperpigmentation over the centrofacial area and extremities was reported in three infants and one child, respectively, after defervescence.

Other than dermatologic, the most involved organ systems with atypical manifestations included gastrointestinal, neurological, and renal systems (Table 1). Acute kidney injury, nausea/vomiting, and meningoencephalitis/encephalopathy were the most common complications for each system, respectively (Table 1). All six out of seven children with encephalopathies or meningoencephalitides developed neurological symptoms within two days of fever onset (Table 2).

**Table 2:** Diagnoses and clinical evaluations of pediatric patients with neurological involvement.

Patient No.	Sex	Age	Diagnosis	Days between fever and neurological onset	CSF Profile						Neuroimaging
					Protein (mg/dL)	Glucose (mg/dL)	CSF/SGlu	RBC (cells/mm^3^)	WBC (cells/mm^3^)	RT-PCR	
1	M	8 y	Meningoencephalitis	2	17	70	0.84	0	2	NE	Normal CT brain
2	M	12 y	Encephalopathy	2	13	67	0.71	1	3	–	Not performed
3	M	8 y	Encephalopathy, septic shock, DIC, AKI, rhabdomyolysis	9	78	45	0.59	18,000	22	–	Not performed
4	F	8 y	Meningoencephalitis	2	44	51	0.56	65	2	+	Normal CT brain and MRI
5	M	7 m	Meningoencephalitis	2	30	71	0.67	67	1	NE	Normal CT brain
6	M	6 d	Congenital CHIKV, TTN, septic shock, hypotonia, OMD, meningoencephalitis	1	80	44	0.49	17,750	14	+	Lenticulostriate vasculopathy from U/S brain
7	M	4 d	Congenital CHIKV, DENV infection, sepsis, OMD, ASD, meningoencephalitis	1	50	61	0.45	1,260	5	+	Cystic changes in bilateral germinal matrix hemorrhage from U/S brain

Abbreviations: AKI, Acute Kidney Injury; DIC, Disseminated Intravascular Coagulation; TTN, Transient Tachypnea of Newborn; OMD, Oromotor Dysfunction; ASD, Atrial Septal Defect; U/S, Ultrasound; CT, Computerized Tomography; MRI, Magnetic Resonance Imaging; CHIKV, Chikungunya Virus; DENV, Dengue Virus; TSS, Toxic Shock Syndrome; CSF, Cerebrospinal Fluid; SGlu, Serum Glucose; RT-PCR, Reverse-Transcription Polymerase Chain Reaction; NE, Not Evaluated; M, Male; F, Female; y, Year; m, Month; d, Day.

Three of the four children with severe, life-threatening presentations had an underlying medical condition (Table 3). All four developed shock (12.9% prevalence overall), and one also had meningoencephalitis. While one had a presentation suggestive of toxic shock syndrome, there was insufficient evidence of other infections after exhaustive investigation other than CHIKV.

**Table 3:** Clinical complications and underlying medical conditions of four children with severe CHIKV presentations.

Case	Sex	Age	Underlying medical condition(s)	Clinical complications
1	M	8 y	Asthma, ASD, left renal agenesis, MPHD	Shock, encephalopathy, rhabdomyolysis, upper GI bleeding, transaminitis, adrenal insufficiency, AKI
2	M	5 y	Asthma, global developmental delay	Shock
3	F	12 y	N/A	Shock, transaminitis, AKI
4	M	6 d	LGA, full-term	Congenital CHIKV, respiratory distress, shock, meningoencephalitis

Abbreviations: LGA, Large-for-gestational-age; ASD, Atrial Septal Defect; MPHD, Multiple Pituitary Hormone Deficiency; GI, Gastrointestinal; AKI, Acute Kidney Injury; CHIKV, Chikungunya Virus; M, Male; F, Female; y, Year; d, Day; N/A, Non-applicable.

### Congenital Chikungunya

Two neonates with CHIKV infections had symptom onsets that occurred within the first seven DOL. Both were full-term but exhibited poor feeding, fever, maculopapular rash, and respiratory distress by the fourth and sixth DOL. Both were plasma/serum and cerebrospinal fluid (CSF) RT-PCR positive. Their mothers had typical, non-severe manifestations of CHIKV infection two days before delivery. Plasma/serum CHIKV RT-PCR testing was positive in one mother on the second day of fever, while the other was not tested. Both neonates had oromotor dysfunction (suck-swallow-breathe incoordination) and required non-invasive respiratory support; one neonate also had hypotonia. During the convalescent phase, both had hyperpigmentation in the centrofacial, periumbilical, and genital areas; desquamation occurred thereafter. Oromotor dysfunction and hypotonia resolved by four weeks of age in one infant and at two months of age for the other. One neonate developed thrombocytopenia on Day 2–3 of fever and had dengue coinfection.

### Investigations

Twenty-five (80.6%) of 31 children had lymphopenia (<1,500/uL), five (16.1%) had anemia, and none had thrombocytopenia (<150,000/uL) at the time of presentation (Table 1). Of the patients tested for alanine aminotransferase (ALT) and aspartate aminotransferase (AST) (n = 21/31 for both), 14.3% (n = 3/21) and 19.0% (n = 4/21) had elevated ALT and AST in ranges of 68–119 U/L and 69–197 U/L. Of the patients with CSF analyses (n = 8/31), 87.5% (n = 7/8) had neurological involvement, and 37.5% (n = 3/8) tested positive for RT-PCR (Table 2). Viral genotyping was performed in six children; all had the E1:K211E/E2:V264A genotype.

### Outcomes

The median (IQR) length of hospitalization overall was 3 (3-7) days. Out of 31 children, two (6.5%) were admitted to the ICU, and five (16.1%) required supplemental oxygen therapy.

At discharge, 13 (41.9%) children (including five with neurological involvement) were fully recovered. Among the remaining children, five (16.1%) still had musculoskeletal conditions, 11 (35.5%) had skin lesions, and two (6.5%) with congenital CHIKV had skin lesions and neurological sequelae, which required orogastric tubes. Follow-ups on the seven children with neurological involvement found that 12.9% (n = 4/7) had normal neurocognitive development 1–3 months after discharge, one had no additional neurological deficits other than a low IQ as an underlying condition, and two had congenital CHIKV. One of these two children was hyperactive but had normal clinical development at 1 year 7 months of age, while the other was diagnosed with delayed speech at 2 years of age. No children lost their lives and all fully recovered from clinical manifestations during hospitalization.

## Discussion

Most hospitalized children at Siriraj Hospital with confirmed CHIKV infection during this outbreak had atypical manifestations despite no underlying medical condition(s). Meningoencephalitides and bullous skin lesions were the most striking atypical presentations. Although a few children had residual neurological deficits, all complications were fully resolved, and none were fatal.

CHIKV was originally considered a self-limiting and non-life-threatening disease before the 2005–2006 outbreak on Réunion Island [6], typically presenting as a fever (2–6 days long) with maculopapular eruptions and other constitutional symptoms [2,17,22–25] (Table 4). After the Réunion island outbreak, severe CHIKV manifestations were increasingly recognized and considered a major public health problem [27]. Only 0.3% of infected adult cases during the Réunion Island outbreak had atypical manifestations (37% had cardiovascular disorders, 24% neurological involvement, 20% pre-renal failure, and 2.8% bullous dermatosis) [6]. Such atypical and severe clinical manifestations may arise from direct or indirect effects of CHIKV infection as well as other coinfections or underlying complications/comorbidities (e.g., hypertension or cardiopulmonary diseases) [2,5]. Despite most adult patients with atypical presentations had underlying medical conditions [6], most children in this study did not. Arthralgia and arthritis were previously reported to be relatively less common in children than adults [17]. Long-term arthritic manifestations, particularly in those that already had compromised joints, may also occur and persist for several months to years [17]. Roughly a third of children in this study had arthralgia, and five children still had musculoskeletal symptoms at discharge. Children may have different clinical manifestations from adults. While pigmentary changes are rare in adults, 42% of children had hyperpigmentation [17]. Hyperpigmentation in the centrofacial area (termed a ‘Chik Sign’) as well as intertriginous aphthous-like ulcers and maculopapular rashes were distinct characteristics of CHIKV infection commonly observed by Ritz *et al.* [17]. Severe dermatological manifestations were also more common in children [2], including vesiculobullous dermatosis, photosensitive hyperpigmentation, and intertriginous aphthous-like ulcers [5,28]. Existing dermatoses (i.e., psoriasis) may also become exacerbated [28]. In a 2016 Indian study, 22% of CHIKV-hospitalized children went into septic shock [24]. Previous studies observed altered liver function and hematology (reduced platelet and lymphocyte counts) during CHIKV infection [2,5,28]. Like previous studies [3,4], this study categorized atypical manifestations based on the affected organ system. Many children developed bullous skin lesions and a few experienced hyperpigmentation after defervescence. Although all children fully recovered at discharge, nearly a 13% prevalence of shock was observed in this study. Also of note, we found a 17.4% of dengue coinfection–a similar prevalence to the 12.4% suspected by Waggoner et al. [13]. This was not unexpected as the same mosquito carrier may harbor both endemic viruses. While elevated transaminase, lymphopenia, and thrombocytopenia are observed in both CHIKV and dengue infections, CHIKV may be further differentiated from dengue and zika by the absence of leukopenia and the presence of arthralgia [12]. Unless clinically indicated by severe or complicated presentations, dengue coinfection is not routinely screened in clinical practice. As there are no specific antiviral treatments for either viral infection, proving coinfection in mild cases may be unnecessary.

**Table 4:** Typical and atypical manifestations and complications of pediatric chikungunya virus infection in the region and overseas.

Author, year	Country	Study design	Findings
Tavares et al. 2017 [22]	Brazil	Single-center, descriptive, cross-sectional study included patients aged 0–17 years hospitalized at Hospital São José and diagnosed with CHIKV fever through positive ELISA IgM in 2017.	Most frequently reported symptoms included fever (n = 42, 100.00%), erythrodermic rash (n = 38, 90.48%), and arthralgia (n = 22, 52.38%). Arthralgia was more prevalent in children >5 years (86.36%, P < 0.05), while vesicobullous rashes were predominant in <5 years (91.67%, P < 0.05). Neurological complications were seen in 6/42 (14.29%) of the patients.
Beserra et al. 2018 [2]	Brazil	Retrospective record review at São José de Doenças Infecciosas, Fortaleza, Ceará from May 2016 to April 2017 which included patients <16 years who presented with a fever and tested IgM positive.	All had a fever (n = 14) which persisted for 5 ± 3 days; 13 (92.8%) had a rash (bullous rash in n = 8); 6 (42.8%) had acute arthralgia (n = 4/6 had polyarthralgia); 2 (14.2%) had meningitis; and 8 (57.1%) had thrombocytopenia.
Naik et al. 2025 [23]	India	Combined retrospective (record review from January 2016 to June 2020) and prospective (from July 2020 to December 2021) study included patients aged 1 month to 15 years who presented with a high fever (>38.5°C for ≥48 hrs) and tested ELISA IgM and/or RT-PCR positive.	Of 58 cases (n = 41 retrospective, n = 17 prospective), 55 (94.8%) had a fever, 32 had skin rash (55.25), 8 (13.8%) had arthralgia, and 5 (8.6%) had seizures. For acute complications, 26 (44.8%) had thrombocytopenia, 11 (19.0%) had AKI, and 4 (6.9%) had seizures.
Sharma et al. 2018 [24]	India	Retrospective, observational study of CHIKV infection confirmed cases (RT-PCR) aged ≤18 years treated at a tertiary care hospital in New Delhi from September to December 2016.	Of the 49 cases included, 13 were severe. Three severe cases had encephalopathy. Commonly observed manifestations included fever (n = 49, 100.0%), rash (n = 23, 46.9%), seizures (n = 12, 24.5%), and arthralgia (n = 8, 16.3%).
Samra et al. 2017 [25]	Honduras	Retrospective, hospital-based chart review of CHIKV (RT-PCR or epidemiologic criteria) confirmed cases aged ≤18 years admitted to Hospital Escuela, Tegucigalpa from January to August 2015.	Of 235 patients, all had fever (100%), 207 had rash (88%), and 49 had arthralgia (21%). For complications, 46% had seizures (n = 24/53) and 39% had meningoencephalitis (n = 21/53).
Rodríguez-Nieves et al. 2016 [26]	Puerto Rico	Retrospective record review of infants born to mothers with chikungunya-like symptoms at the University Pediatric Hospital, UPR Carolina Hospital and San Juan City Hospital, Neonatal Intensive Care Units from August 2014 to January 2015.	Of 10 infants, 7 were born to mothers who had symptoms ≤5 days from delivery–with 40% and 30% reporting fever and thrombocytopenia, respectively; 3 were born to mothers who had symptoms >5 days after delivery–all with congenital anomalies (e.g., hydrocephaly, brain infarcts).
Carillo et al. 2025 [12]	Nicaragua	Single-center prospective cohort study which included laboratory-confirmed cases of dengue, zika, and CHIKV who enrolled in their Pediatric Dengue Cohort Study between January 19, 2006, and December 31, 2023, and were evaluated at the Health Center Sócrates Flores Vivas.	Most common clinical features of CHIKV patients were fever (n = 517/517, 100%), arthralgia (n = 446/517, 86.3%), lymphocytopenia (n = 441/517, 85.6%), and headache (n = 396/517, 76.6%). Arthralgia was most prevalent on Days 1–4.

Abbreviations: AKI, Acute Kidney Injury; CHIKV, Chikungunya Virus; ELISA, Enzyme-linked Immunosorbent Assay; RT-PCR, Real-time Polymerase Chain Reaction.

Despite mortality rates of CHIKV infection being low (1 in 1,000 clinical cases during the Réunion Island outbreak [29]), elderly populations, individuals with underlying medical conditions (i.e., diabetes mellitus, hypertension, or cardiovascular disease), as well as neonates with/without intrapartum exposure, have an increased risk of severe disease [5,6,17,27]. Congenital CHIKV was first reported during the Réunion Island outbreak with a transmission rate of 48.7% [7]. Similar rates of 27.7–48.3% were observed in Latin America [30]. All neonates with symptomatic presentations were born to mothers with intrapartum CHIKV infection, with a median (range) onset of neonatal illness of 4 (3–7) days [5,17]. While maternal CHIKV infection during early stages of pregnancy does not appear to lead to symptomatic presentations in neonates [31], an increased risk of neonates developing congenital CHIKV was reported in CHIKV viremic mothers a week before delivery – with the greatest observed risk being two days before or after delivery [17,31]. Full-term newborns in this study and Rodríguez-Nieves *et al.*’s [26] with congenital CHIKV developed symptoms within this range. Previous studies reported 68.7% of neonates had encephalopathy or encephalitis, and 44.7% of neonates developed hypotonia [7,32]. Two neonates in our study were diagnosed with meningoencephalitis with positive CHIKV RT-PCR for CSF. Both neonates had sepsis-like illness, fever, poor feeding, irritability, limb edema, and rash. Neonates with CHIKV-infected mothers should be monitored for at least seven days after birth and followed-up on at least two years thereafter to monitor possible neurological sequelae [31].

Para- and post-infectious neurological involvement of CHIKV infection is wide-ranging in manifestation, for example Guillain-Barré syndrome, meningoencephalitis, myelopathy/myelitis, encephalopathy/encephalitis, myeloneuropathy, neonatal hypotonia, neuro-ocular diseases, and more [5,9,31]. This involvement is consistently one of the most reported complications from CHIKV across both children and adults [31]. It may also be more significant in children than previously documented, with a 40–50% prevalence of severe neurological manifestations (e.g., complex seizures, epilepticus, and encephalitis) and 14–32% prevalence of encephalopathy, seizures, and meningoencephalitis [17]. During the Réunion Island outbreak 25% of children with laboratory-confirmed CHIKV infection presented with neurologic manifestations, 10% had encephalitis, and two died [11]. A 11% prevalence of meningoencephalitis in children with RT-PCR confirmed CHIKV was reported in a Honduras study; one child with severe meningitis died [25]. We observed a similar prevalence of meningoencephalitis and encephalopathy during acute infection. Long-term sequelae and disabilities from neurological complications are often reported across adults and children, particularly neonates [5,17]. Up to two years after acute infection, 75% of patients with atypical neurological manifestations experienced attention and memory difficulties and 50% experienced sensorineural disorders (i.e., blurred vision, hearing difficulties, etc.) [17]. During the Réunion Island outbreak, 50% of children with neurological manifestations reportedly had neurodevelopmental delays at two years of age [33]. Most of the CHIKV patients with neurological involvement in this study recovered at discharge, only two had neurological sequelae. Previously recorded neurological sequelae across other encephalitic alphaviruses (Western and Eastern equine encephalitis viruses, Venezuelan equine encephalitis virus), including: convulsions, seizures, altered mental status/personality, and/or intellectual disabilities [34]. This highlights the importance of long-term follow-ups with children who are suspected of having neurological involvement.

There are three distinct CHIKV genotypes, namely Asian, Western African, and East/Central/South African (ECSA). The Indian Ocean Lineage (IOL) arose from the ECSA lineage and has caused several epidemics since 2005 in South and Southeast Asia, the Indian Ocean Islands, and Europe. These different viral genotypes may explain variations in clinical manifestation and severity. Asian genotypes may have milder presentations than those caused by ECSA genotypes [35]. During the 2014 Caribbean outbreaks of an Asian CHIKV genotype, no severe complications–except for one case of myocarditis with dengue coinfection–were observed in infected children in Barbados [36]. However, this genotype also caused febrile seizures in 17.6% of pediatric CHIKV cases in Jamaica [37]. The circulating clade on Réunion Island during the 2005–2006 outbreak was ECSA-IOL, specifically the ECSA strain with the E1:A226V genotype–which was described to cause more severe and atypical manifestations [38,39]. In contrast, the large 2008–2009 and 2013 outbreaks in Thailand–caused by the same strain and genotype–were not commonly associated with atypical or severe disease [40]. The 2019 outbreak in Thailand was driven by a mutated ECSA strain with the E1:226A genotype (E1:K211E/E2:V264A), which enhanced viral infectivity, dissemination, and transmission in *Aedes aegypti* mosquitoes [41,42]. All six children in this study who underwent viral genotyping were confirmed to have been infected with this strain and genotype. Four and seven cases included in our study had severe presentations and neurological manifestations, respectively, with possible long-term neurological complications. This mutated strain was also documented in outbreaks in India (2015–2017) [43], Pakistan (2016) [44], Bangladesh (2017) [45], and Italy (2017) [46]. Altogether, genotypic features of CHIKV strains may partly account for the rise in atypical and severe manifestations.

It is crucial that medical practitioners maintain a high index of suspicion for CHIKV infection in endemic areas to ensure early diagnosis and appropriate treatment to minimize complications and improve clinical outcomes. Further research assessing differences in severity and frequency of atypical manifestations between patients infected with parental and mutant strains is warranted.

### Limitations and future research

This study had some limitations. First, it included <18 years old patients hospitalized at a single tertiary care center. This information may not be applicable to other ages or demographic groups in other settings. Second, this study was vulnerable to selection and recollection bias due to its retrospective design. Third, it included and analyzed a small sample size. This stems from the limited data of infected children in the region during this recent outbreak and underscores the increased severity of re-emerging CHIKV infections. This study helps to address this knowledge gap. The epidemiological findings discussed in this study can be further explored and validated through future multicenter trials with large cohorts.

## Conclusions

After a 60-year hiatus, CHIKV re-emerged in Bangkok in 2019. Unlike previous outbreaks, atypical manifestations and severe presentations were common during this outbreak, despite most children in our cohort having no underlying medical condition(s). Although the cohort is small, the observed frequency of neurological complications attributed to CHIKV infection justifies long-term follow-up in children with neurological manifestations and complications. CHIKV should be suspected and tested for in febrile children, particularly those with rashes and neurological involvement, in endemic countries like Thailand.

## Supporting information

S1 Appendix Raw data of included patients. (XLSX)
